# Beyond numbers: challenges in assessing myasthenia gravis prevalence in inflammatory bowel disease

**DOI:** 10.1055/s-0045-1815739

**Published:** 2026-03-23

**Authors:** Christian Messina

**Affiliations:** 1 Azienda Sanitaria Provinciale di Catania, Catania, Italy.

Dear Editor,

We read with great interest the article by Leitão et al., which investigated the association between myasthenia gravis (MG) and inflammatory bowel disease (IBD). 1 In a Brazilian cohort of 606 IBD patients, MG was identified in 4 individuals (0.66%), including 1 with ulcerative colitis (UC) and 3 with Crohn's disease (CD). 1 Notably, 2 patients were acetylcholine receptor antibody positive, 1 was seronegative, 1 was anti-muscle specific kinase positive—the first such report in this context. 1 Abnormal repetitive nerve stimulation was observed in three patients, while nerve conduction studies were normal; all exhibited mild small fiber neuropathy (SFN). 1 The prevalence of MG in IBD patients was significantly higher than in the general cohort at the authors' center, highlighting a notable overrepresentation. 1 However, despite the novelty and relevance of these findings, several methodological limitations and points requiring clarification warrant discussion.

A major limitation of the study is the lack of detailed remote medical and pharmacological history for the reported patients. Several medications are known to trigger the onset of MG, so without this information, it is difficult to attribute MG directly to IBD. 2 Of particular importance are immune checkpoint inhibitors, which are standard-of-care treatments for a variety of metastatic cancers and can induce multiple autoimmune disorders, including MG. 2

Similarly, D-penicillamine has been associated with the development of autoimmune conditions, including MG. 2 This drug is used in the management of autoimmune diseases such as rheumatoid arthritis, primary biliary cirrhosis, and scleroderma, as well as in Wilson's disease and cystinuria. 2 Other agents, including alemtuzumab, tyrosine kinase inhibitors (TKIs), and interferons, may also trigger autoimmune manifestations. 2 Therefore, without a thorough review of prior therapies, it remains uncertain whether MG in these patients is truly associated with IBD, or rather represents a drug-induced autoimmune event not captured in the study.

Another important limitation of the study is that MG investigations were not systematically performed across the entire cohort of 606 IBD patients. Consequently, the true prevalence of MG in this population remains uncertain. Several factors may contribute to this potential underestimation or misrepresentation. Some patients could have been lost to follow-up, leaving their clinical status unknown. Others may have developed only mild symptoms, or their manifestations may have been suppressed by corticosteroids or other immunosuppressive therapies. Conversely, it is also possible that the patients identified as MG-positive experienced symptom exacerbation due to exposure to certain medications, 2 such as antibiotics used for IBD-related infections, while others who have not yet received such triggers may remain asymptomatic. This introduces a clear sampling bias, suggesting that the actual prevalence of MG in IBD patients could be higher than reported.

Furthermore, it is important to address the issue of small fiber neuropathy (SFN) reported in the study. The diagnostic approach used—namely the skin wrinkling test—does not conform to internationally recognized criteria for SFN, raising concerns about the validity of these findings. 3 Moreover, as previously discussed, the lack of detailed remote medical and pharmacological history makes it difficult to establish a true association between SFN and IBD or MG. Given how SFN can result from a wide range of medical conditions, medications, or even vaccinations, 4 without a comprehensive evaluation, the observed cases may reflect secondary or unrelated causes rather than a direct disease-specific association.

In conclusion, while the study by Leitão et al. 1 provides valuable insights into the potential association between MG and IBD, several methodological limitations—including incomplete patient histories, selective testing, and nonstandardized diagnostic criteria for small fiber neuropathy—warrant cautious interpretation of the findings. Addressing these issues in future studies will be essential to accurately characterize the prevalence and mechanisms of MG in IBD, disentangle disease-specific factors from drug- or lifestyle-related ones, and ultimately improve patient care in this complex population.
